# Factors associated with receipt of informal care among the Vietnamese older persons: Evidence from a national survey

**DOI:** 10.3389/fpubh.2023.1065851

**Published:** 2023-03-08

**Authors:** Long Thanh Giang, Nam Truong Nguyen, Tham Thi Hong Pham, Phong Manh Phi

**Affiliations:** ^1^National Economics University, Hanoi, Vietnam; ^2^Institute of Social and Medical Studies, Hanoi, Vietnam; ^3^Hanoi University of Mining and Geology, Hanoi, Vietnam

**Keywords:** activities of daily living (ADLs), aging, care providers, older people, Vietnam, informal care

## Abstract

**Background:**

The rapidly growing older population in Vietnam poses an increasing need for care among the older persons, who have mainly relied on informal care at homes and communities. This study examined the Vietnamese older persons' individual and household factors determining their receipt of informal care.

**Methods:**

This study provided cross-tabulations and multivariable regression analyses to identify who provided assistance to the Vietnamese older people along with their individual and household characteristics.

**Data:**

The nationally representative survey on older persons, namely Vietnam Aging Survey (VNAS) in 2011 was used in this study.

**Results:**

We found that proportions of older persons having difficulty in activities of daily living (ADLs) were different in regard to their age, sex, marital status, health status, working status, and living arrangements. In care provision, gender differences were clear, in which females generally had significantly higher rates of providing care to older persons than their male counterparts.

**Conclusion:**

Care for older persons in Vietnam has been mainly provided by their families, and thus changes in socio-economic, demographic factors along with differences among generations in family values will be a key challenge to maintain such care arrangements.

## Introduction

The world has faced declining fertility rates and increasing life expectancies, which have resulted in an aging population, and this demographic trend is considered one of the most critical socio-demographic challenges in the twenty-first century (1, 2). The population projection by United Nations Department for Economic and Social Affairs—UNDESA (3) showed that the proportion of older people (defined as those aged 60 and older) in the world would be doubled by 2050 (from around 12% in 2018 to about 25% in 2050), and about two-thirds of them would live in low- and middle-income countries (LMICs).

Population aging implies various socio-economic and health issues in both developed and developing countries. A number of studies have shown that aging is strongly related to increasing health problems, resulting in higher risks of disability and chronic diseases. Consequently, demand for care of older people is on the rise (4–6). Care for older persons has become an emerging challenge in LMICs, as healthcare delivery systems and policy-related care for older people in these economic regions are still underdeveloped to cope with such a rapid demographic transition (4, 7).

Vietnam is no exception to the above-mentioned demographic trend. In 2019, there were about 11.4 million older persons (or about 11.9% of the country total population) (8). Specifically, it is projected that the Vietnamese older people will account for about 25% of the total population by 2050 (9). Among older population, the oldest-old (defined as those aged 80 years or over)—who are most likely to need both health and social care—will increase at the highest rate, and older women will continue to be predominant, which shows a clear trend of “feminization of aging” (10, 11). In regard to care for older people, Vietnamese families are still playing a key role in providing care for their old-aged parents and older generations, while hospitals and other institutional care providers are limited in terms of elderly care services (12). However, such family care has faced various issues: family size is reducing to one with fewer number of children; multi-generational co-residence is turning to nuclear co-residence with an increasing rate of older couples living together only; and family members are quickly dispersed due to rapid urbanization along with a large outmigration of rural youth to urban cities (11, 12). More particularly, the rate of older persons living together with at least one child decreased substantially from 85% in early 1990 to merely 40% in 2016. Consequently, the rate of “skipped-generation” families (where great/grandparents live with only great/grandchildren) has increased significantly in rural areas (13–15). As Vietnam's long-term care system is just in the early stage of development, family/home-based care has been the main source of care for older persons (12).

Given such critical milestones for aging population and care needs, it is necessary to explore the current situation of informal care provision to the older persons and its determinants in order to provide appropriate policy implications to the Government of Vietnam and its line ministries in design and implementation of care programmes. To date, most of the existing studies have discussed care provision for older persons with focuses on living arrangements and cash or in-kind support [see, for instance (16, 17)]. Moreover, majority of the existing studies used small-scale or unrepresentative data of older population, so their findings could not reflect different factors of care-giving decisions to older persons. For instance, Tran (17) analyzed the association between living arrangements and providing care for older people living in rural areas in Vietnam, using data from ChiliLab—a longitudinal data on older persons living in a rural commune in Hai Duong province. The study found that most older people were living in multi-generational households due to financial dependence, and that care for older persons was provided in exchanging with childcare, i.e., older persons took care of their grandchildren. Vietnam's Women Union (18), using data collected from a commune in Thai Nguyen province, analyzed the living conditions of older people who had children migrating to other provinces. The research showed that older people were the main caregivers of their grandchildren, and that they were taken care of mostly by their spouse or neighbors. To date, only VWU (18) provided cross-tabulation statistics on care for the elderly without in-depth analyses on its association with the possible underlying factors.

Therefore, the current study, using a national survey on the Vietnamese older people, namely Vietnam Aging Survey (VNAS), filled such a research gap by providing analyses of care needs related to older people's limitations in activities of daily living (ADLs) as well as factors determining their received care. In particular, this study explores the needs of care among the Vietnamese older persons based on their status of ADLs, and by whom an older person is provided care, and then analyses factors associated with their receipt of informal care. Compared to previous researches, this study provided more a comprehensive construction of variables which more deeply reflected social, economic and health conditions of the Vietnamese older people.

This research was aimed to answer the following questions: (i) Which groups of the older persons needed care? (ii) How many percent of the older persons in need received care? (iii) Who provided informal care to older persons? and (iv) What were the factors determining older people's receipt of care?

## Data and methods

### Data

In order to answer the above research questions, we used the Vietnam Aging Survey (VNAS) in 2011, which was as the first nationally representative survey on people aged 50 years and over in Vietnam (18). To date, there have been two more surveys; however, they are not publicly available so that we could not use them. The VNAS 2011 was conducted with the approval from the Institutional Review Board in Biomedical Research Number 308/Hððð-ISMS dated 09 May 2011 at the Institute of Social and Medical Studies (ISMS), Hanoi, Vietnam.

The sample of VNAS was chosen by a multi-stage sampling framework with the data of the 2009 Population and Housing Census. The sampling method used was probability proportional to size (PPS), which was the most common method used in household surveys to select a sample in each stage of the sampling design. VNAS sampling had four steps, including: (i) selected 12 provinces from six ecological zones; (ii) chose 200 communes from 12 selected provinces; (iii) randomly selected 2 villages from each selected commune; and (iv) in each village, randomly took 15 people aged 50 years old and above for interviews, in which 10 people were officially interviewed and 5 people were reserved as alternatives.

VNAS collected various pieces of information on demographic, social, economic, and health characteristics of the surveyed people as well as their household situations (such as housing and assets). Data were collected by personal interviews. All interviewees or their legal representatives had to express their consent to participate in the interviews. The interviews were conducted in private to ensure confidentiality and privacy. The identities of all the interviewed participants and the recorded information on the questionnaire about their relatives, as well as the analysis data were encrypted and kept confidential.

The final VNAS sample included 4,007 persons aged 50 and over, in which there were 2,798 older persons (those aged 60 and over).

## Methods

### Statistical analysis

For the first three research questions, descriptive analysis was conducted to report the older population who needed care and those who received care, with different background characteristics. Chi-square test was used to examine the statistical significance of the associations between these background characteristics and care need and care receipt of older people.

For the last research question, a multivariate logistic regression model was performed to adjust for the possibly confounding impacts of different variables (presented below). The effects of these exploratory variables on the dependent variable were measured by their odds ratios (OR). A *p*-value of <0.1 was regarded as statistically significant.

To check multi-collinearity among independent variables, we applied Variance Inflation Factor (VIF) for all independent variables, and only kept variables with values of VIF being smaller than 4 as the popular “rule of thumbs” (19). To check the goodness-of-fit of logistic regression model, we used Hosmer-Lemeshow test (20).

A number of studies [see, for instance (12, 21, 22)] indicated that urban and rural older persons were different in health conditions and care needs, and therefore we used Chow test to examine whether regression coefficients estimated for the rural sample were significantly different from those for the urban sample. If that was the case, we would run separate logistic regression models for rural and urban areas; otherwise, we would run logistic regression model for the full sample only.

In all calculations, we used the sample weights to make all results representative for the whole older population, using Stata command “svy.”

### Variable description

Based in the analytical frameworks developed by Vlachantoni et al. (23), there are three groups of the potential factors affecting the receipt of informal care by older persons: need factors; demographic factors; and socio-economic factors. As discussed in various studies [such as (6, 24–26)], need for care has been measured by levels of difficulty in performing activities of daily living (ADL) or instrumental activities of daily living ADL (IADL), or by health-related factors (such as severity of physical disability, self-rated health, or psychological health). The demographic factors (such as age, gender, marital status, and living arrangements) are also important determinants of care receipt by older persons. For instance, Pickard et al. (27) and Teerawichitchainan and Knodel (26) found a strong association between care receipt and older people's living arrangements, while Glaser et al. (28), Vlachantoni et al. (23), and Phi et al. (29) showed that marital status was significantly associated with care receipt. The socio-economic factors have played a role in determining care receipt by older people, but its role has not been consistent in different country contexts. For example, older persons with higher income levels had lower probability to receive informal care (24, 30). Educational qualification was strongly associated with care receipt for older persons in Myanmar (26), but not for those in Vietnam (29).

Based on the above review as well as availability of the VNAS 2011, below are variables in our consideration.

#### Care need

In this study, an older person was considered to need care when he/she had at least a difficulty in any ADL. In the VNAS 2011, the construction of ADLs was based on five vital activities in daily living, which were: “eating,” “getting dressed and undressed,” “bathing/washing yourself,” “getting up when you are lying down,” and “getting to and using the toilet.” Responses for each activity consisted of four levels of difficulty: “No difficulty at all,” “A bit difficult,” “Moderate,” “Very difficult,” and “Could not do any ADL.” Older respondents who answered “No difficulty at all” for all ADLs were considered as persons with “No difficulty” in ADLs, and they were coded 0 (i.e., they did not need care), while the other persons who had other answers were considered as those who “Had at least one difficulty in ADLs” and they were coded 1 (i.e., they needed care).

#### Care receipt and care providers

In regard to care for older people, respondents were asked by the following question: “When it comes to doing things you need to do to take care of yourself, like bathing and getting dressed, do you receive any help from anyone?,” and there was a binary answer, i.e., “Yes” or “No.”

To explore from whom the older respondents received assistance, they were asked by the following question: “Can you tell me who helps you?,” and the possible responses included “Spouse,” “Son,” “Daughter,” “Son in law,” “Daughter in law,” “Grandson,” “Granddaughter,” “Other relatives,” “Community members/neighbors/friends,” “Hired workers/care-givers,” “Health workers,” and “Other persons.”

#### Independent variables

Demographic variables included:

- *Age* included three groups: those aged 60–69 (or the young-old); those aged 70–79 (or the middle-old), and those aged 80 and over (or the oldest-old).
- *Sex* included older men and older women.
- *Marital status* included two groups: (i) currently married; and (ii) currently unmarried (e.g., single, divorced, separated, and widowed).

Health-related variables included:

- Self-rated health (SRH) was measured as a binary variable, which was based on its origin of a Likert scale of five points: very poor; poor; fair; good; and very good. Those who answered “good” or “very good” were considered as those with “good SRH,” while those with the remaining answers were considered as those with “bad SRH.”
- Psychological distress symptoms were based on the following symptoms in the past week prior to the survey: “Did not like eating and appetite was poor,” “Felt sad or depressed,” “Had difficulty in sleeping,” “Felt unhappy,” and “Felt lonely.” Those who answered “Some of the time” or “Most of the time” for all these symptoms were considered as those with depression, while those who answered “Not at all” for all these symptoms were considered as those without any depression.

Socio-economic and household-related variables included:

- *Education* was presented by the readability of an older person. Each was asked the question: “Do you know how to read?,” and a respondent could choose one from “No,” “Yes, but with difficulty,” “Yes, easily,” and “I used to but forgot.” In the analyses, this variable was categorized into two groups, including one for those who answered “Yes, easily” (considered as those with “Able to read easily”), and one for the other (considered as those with “Unable to read easily”).
- *Employment status* included two groups—one for those who were working, and the other for those who were not working at the time of the survey conducted.
- *Place of residence* included urban and rural areas.
- *Living arrangements* included three groups: lived alone; lived with spouse only; and lived with others (children, grandchildren, relatives, etc.).
- *The household wealth index* was constructed following Vyas and Kumaranayake (31), using principal components analysis (PCA) method. The wealth scores were constructed by household durable assets (such as motorbikes, cars, and phones), housing quality (such as materials of roof and floor) and sanitation facilities (such as sources of drinking water and type of toilet). From the final wealth scores, five quintiles were constructed, in which the first quintile and the fifth indicated the poorest and the richest, respectively. In our analyses, households in the first and second quintiles were considered those with the “poor wealth” status; those in the third quintile were considered the “average wealth” status; while those in the fourth and fifth quintiles denoted those with the “rich wealth” status.
- *Household ownership* was divided into three groups: owned by older person or spouse; owned by children/children-in-law; and owned by others.

## Results

### Characteristics of older people with difficulty in ADLs and care receipt

Table 1 provides the descriptive results for the difficulty in ADLs of the Vietnamese older people along with their characteristics.

**Table 1:** Percentage of older people with at least a difficulty in any ADL.

**Characteristics**		**At least a difficulty in any ADL (*N* = 2,789)**	***p*-value**
**Total**		**37.11**	
**Demographic characteristics**			
Age groups			<0.001
	60–69	26.45	
	70–79	41.48	
	80 and over	52.22	
Sex			<0.10
	Female	39.83	
	Male	33.27	
Marital status			<0.05
	Currently married	34.65	
	Currently not married	41.94	
**Health characteristics**			
Self-rated health			<0.001
	Good	17.27	
	Bad	48.20	
Distress symptoms			<0.05
	No symptom	30.46	
	At least one symptom	38.60	
**Socio-economic and household-related characteristics**			
Educational level			<0.001
	Unable to read	47.98	
	Able to read easily	28.70	
Currently working?			<0.001
	No	43.30	
	Yes	27.32	
Living area			<0.01
	Rural	40.35	
	Urban	30.50	
Living arrangements			<0.05
	Living alone	49.15	
	Living in a couple	32.99	
	Other	37.66	
Household's wealth			0.2082
	Poor	33.95	
	Average	38.45	
	Rich	41.56	
House ownership			<0.01
	Older person or spouse	34.76	
	Children/children-in-law	56.23	
	Other	36.72	

p-values were from χ^2^ tests for all categorical variables.

Source: Own calculations, using data from VNAS 2011.

In general, 37.11% of the Vietnamese older people had at least one difficulty in ADLs. The prevalence of difficulty in ADLs was significantly higher for more advanced age persons (*p* < 0.001); higher for women than for men (39.83% vs. 33.27%; *p* < 0.1); higher for the unmarried than the married (41.94% vs. 34.65%; *p* < 0.05); higher for those with bad self-rated health (*p* < 0.001), and higher for those with psychological distress symptoms (*p* < 0.05). Regarding socio-economic and household-rated characteristics, there were statistically significant differences in readability (*p* < 0.001), working status (*p* < 0.001), place of residence (*p* < 0.05), living arrangements (*p* < 0.05), and house ownership (*p* < 0.01).

Table 2 shows the weighted prevalence of receiving care by older persons who faced at least a difficulty in ADLs, by their characteristics.

**Table 2:** Percentage of receiving care in any ADL by older persons.

**Characteristics**		**%Received care**	***p*-value**
**Total**		38.46	
**Demographic characteristics**			
Age groups			<0.001
	60–69	27.37	
	70–79	29.71	
	80 and over	58.60	
Sex			0.9324
	Female	38.67	
	Male	38.14	
Marital status			0.6650
	Currently married	37.55	
	Currently not married	40.10	
**Health characteristics**			
Self-rated health			<0.001
	Bad	43.18	
	Good	15.54	
Distress symptoms			<0.001
	No symptom	74.70	
	At least one symptom	32.17	
**Socio-economic and household-related characteristics**			
Educational level			0.1175
	Unable to read	42.18	
	Able to read easily	33.32	
Currently working?			<0.001
	No	48.26	
	Yes	14.46	
Living area			0.1757
	Rural	36.08	
	Urban	45.05	
Living arrangements			<0.10
	Living alone	22.46	
	Living in a couple	33.47	
	Other	39.59	
Household's wealth			0.7389
	Poor	36.18	
	Average	40.72	
	Rich	36.80	
House ownership			<0.05
	Older person or spouse	36.37	
	Children/Children-in-law	51.97	
	Other	21.87	

p-values were from χ^2^ tests for all categorical variables.

Source: Own calculations, using data from VNAS 2011.

The results showed that the proportion of older people receiving assistance was consistently and significantly increased with age, especially among the oldest-old group (58.60% for those aged 80 and over, compared to 29.71% for those aged 70–79 and 27.37% for those aged 60–69, *p* < 0.001).

Self-rated health showed statistically significant differences between older groups. Those reporting bad self-rated health had a higher rate of receiving care than their respective counterparts (43.18% vs. 15.54%, *p* < 0.001). Interestingly, among those with/without distress symptoms, however, the situation was different: those having no distress symptom had statistically significantly higher rate of receiving care than those having at least one symptom (74.70% vs. 32.17%, *p* < 0.001).

For employment status, working older persons had a significantly lower rate of receiving care than did non-working older persons (14.46% vs. 48.26%, *p* < 0.001).

In terms of living arrangements, older people living alone had a statistically significantly lower proportion to receive care than did older people with other arrangements. Similarly, older persons living in households which were owned by their children/children-in-law had a very high rate of receiving care than did those living in other types of house ownership.

There was no statistically significant difference in rate of receiving care between older people in terms of sex, marital status, readability, place of residence and household's wealth.

### Who provided care to older Vietnamese with at least a difficulty in ADLs?

Table 3 presents the percentage of older people who had at least a difficulty in ADLs and received care from different persons. It is important to note that an older person could receive care from many persons at the same time, so the total percentage presented in each row in Table 3 was not necessary to be 100%.

**Table 3:** Comparison between older groups by their characteristics on proportions of receiving care from various care-givers.

**Characteristics**		**Spouse**	**Son**	**Daughter**	**Son-in-law**	**Daughter-in-law**	**Grandson**	**Grand-daughter**	**Hired care-givers**	**Other**
**Total**		**42.44**	**37.04**	**43.13**	**6.14**	**34.67**	**8.30**	**19.38**	**1.92**	**0.50**
**Age groups**										
	60–69	80.84^***^	31.87	35.79^*^	3.63	17.31^*^	0.84	13.83	0.00	0.05^*^
	70–79	55.42	42.67	29.84	7.39	33.85	12.96	19.85	0.00	0.66
	80 and over	17.20	36.33	53.70	6.60	43.18	9.17	21.71	3.87	1.19
**Sex**										
	Female	24.33^***^	30.88^+^	49.23^*^	4.75	38.20	10.46	27.35^***^	3.09	0.69
	Male	71.82	47.10	32.91	8.40	28.91	4.77	6.41	0.01	0.27
**Marital status**										
	Currently not married	0.00	39.64	62.41^***^	11.29^*^	46.29^*^	11.40	25.17	0.92	1.16^*^
	Currently married	67.62	35.50	31.44	3.06	27.72	6.44	15.93	2.50	0.20
**Living area**										
	Rural	40.81	36.17	36.63^*^	3.10^*^	37.24	8.82	18.64	0.01	0.74^***^
	Urban	45.79	39.04	57.54	13.02	28.81	7.11	21.07	2.76	0.02
**Living arrangements**										
	Living alone	0.00^***^	31.39^*^	37.12^**^	22.88^*^	46.92^*^	17.14	40.94^**^	0.00	2.16^*^
	Living with spouse	86.87	20.80	18.44	4.06	19.20	5.52	5.28	0.01	0.22
	Other	33.54	43.93	47.41	6.14	40.14	8.93	19.68	0.46	0.48

*** p < 0.001;

** p < 0.01;

* p < 0.05;

+ p < 0.1. The p-values were from χ^2^ tests for all categorical variables for each type of care-givers.

Source: Own calculations, using data from VNAS 2011.

The results were quite similar to those presented in Table 2. Across older persons' characteristics, however, there were noticeable gender differences of care-givers. More particularly, females usually had significantly higher rates of providing care than did their male counterparts. In particular, wives, daughters, daughters-in-law, and granddaughters had significantly higher rates of providing caregiving for older persons than did husbands, sons, sons-in-law, and grandsons. For example, 71.82% of older men were cared by their wives, while only 24.33% of older women were cared by their husbands. The oldest-old group was mostly cared for by their daughters, daughters-in-law, and grand-daughters, but they had the lowest rate of being cared for by spouse—partly because of their highest rate of widowhood.

### Factors associated with care receipt among older persons with at least a difficulty in ADLs

As mentioned earlier, to identify factors determining the received care of older persons, we conducted multivariable logistic models. To determine whether we needed to conduct separate models for older persons living in urban and rural areas or a pooled model for the full sample, we used Chow tests (32). The sample included 739 urban older persons, and 2,050 rural older persons. With the chosen independent variables, the Chow test showed $\chi_{\left(13\right)}^{2}$ = 16.23; Prob > χ^2^ = 0.2318, meaning that the null hypothesis (i.e., urban and rural older persons were not statistically significantly different from each other in terms of receiving care in ADLs) was not rejected. Thus, we used the full sample for multivariable analysis and did not consider rural-urban stratification models.

Results for VIF show that VIF value for each variable was smaller than 4, suggesting no evidence of multicollinearity among independent variables used in this study. Therefore, all variables were controlled in multivariable regression analysis. With these independent variables, we applied the Hosmer-Lemeshow test for the goodness-of-fit, and results indicated no evidence of lack of fit (*p* = 0.652).

Table 4 shows the results of the logistic model, presenting factors determining care receipt by older persons who had at least one difficulty in ADLs.

**Table 4:** Factors determining care receipt by older people.

**Independent variables**	**Adjusted OR**	**90% CI**	
		**Lower**	**Upper**
**Demographic factors**			
**Age groups**			
60–69 (ref.)	1.00		
70–79	1.16	0.74	1.80
80 and over	2.86^***^	1.95	4.20
**Sex**			
Female (ref.)	1.00		
Male	0.96	0.70	1.33
**Marital status**			
Currently unmarried (ref.)	1.00		
Currently married	2.11^***^	1.39	3.20
**Health-related factors**			
**Self-rated health**			
Bad (Ref.)	1.00		
Good	0.11^***^	0.06	0.19
**Psychological distress symptoms**			
At least one symptom (Ref.)	1.00		
No	0.36^***^	0.25	0.53
**Socio-economic and household-related factors**			
**Educational level**			
Unable to read (ref.)	1.00		
Able to read easily	0.70	0.49	1.02
**Employment status**			
Currently not working (ref.)	1.00		
Currently working	0.26^***^	0.17	0.40
**Place of residence**			
Rural (ref.)	1.00		
Urban	0.95	0.61	1.47
**Living arrangements**			
Alone (ref.)	1.00		
With only spouse	0.49^+^	0.25	0.94
With other	0.65	0.38	1.10
**Household's wealth**			
Poor	1.00		
Average	1.60	0.99	2.61
Rich	1.09	0.67	1.77
**House ownership**			
By other (Ref.)	1.00		
By older person or his/her spouse	0.85	0.54	1.32
Constant	0.55	0.24	1.26
*F*_(14, 386)_ = 13.76; Prob > *F* = 0.0000			

Ref.-reference category;

*** p < 0.001;

^**^p < 0.01; ^*^p < 0.05;

+ p < 0.1.

Source: Own calculations, using data from VNAS 2011.

The oldest-old group—who had the highest rate of having at least one difficulty in ADLs—had nearly three times higher possibility of receiving care than young-old groups (*p* < 0.001). Older persons reporting good self-rated health were about 89% [100^*^(1–0.11)] less likely to receive care than their counterparts who reported bad self-rated health (*p* < 0.001). Similarly, people without psychological distress symptoms were 64% [100^*^(1–0.36)] less likely to receive care than those who had at least one symptom (*p* < 0.001). These results were consistent with those found for older persons in other countries [see, for instance, (33) for Americans; (34) for Chinese; (35) for Australians]. Also, working older persons had about 74% [100^*^(1–0.26)] less likely to receive care than non-working counterparts (*p* < 0.001), and this finding was the same as that for Indonesian older persons (36) and for Australians (35).

The married persons had about two times higher possibility to receive care in ADLs than the unmarried counterparts, and this could be explained by the fact that the former usually have more children and grandchildren than do the latter so that they have more opportunities to be provided care.

Regarding living arrangements, an older person living with spouse only had about 51% [100^*^(1–0.49)] lower likelihood to receive care than those living alone. In fact, older persons living as couples are usually those who could do ADLs quite well by themselves, so that they do not need to be taken care of by others.

Other factors, including sex, readability, place of residence, household's wealth, and house ownership, did not establish their significant associations with the probability of receiving care among older persons with at least one ADL difficulty.

## Discussion

This study described the prevalence of having at least one ADL difficulty among the Vietnamese older people, and explored factors determining their possibility of receiving care.

The results indicated that advanced ages and health conditions were significant factors in presenting ADL difficulty and determining care receipt of older people. More importantly, among caregivers, the results showed that wives, daughters, and granddaughters played a crucial role in assisting older Vietnamese in carrying out ADLs. Such family care arrangements could be attributed to traditional filial piety in Vietnam as well as mandates in various constitutions and laws (such as all Constitutions from 1946 to 2013, Law on Marriage and Family, and Law on the Elderly). Also, gender differences—especially between older couples taking care of each other—could be supported by the fact that in the Vietnamese culture, women are generally more likely to marry men at older ages as well as that women tend to live longer than men (18, 37, 38). As a result, husbands usually need care earlier in their married life and have spouse to provide such care (12, 39). Ironically, when older wives have sickness and need care, they might be more likely to be widowed and at risk of care shortage (33, 39, 40).

In addition to receiving care from spouse and children, as mentioned earlier, this study also indicated that grandchildren, especially granddaughters, are also providing care to their grandparents. Earlier studies on living arrangements of the Vietnamese older people [such as (13, 14, 41)] showed that older people living in multi-generational households, particularly skipped-generation ones, were beneficial to their (great)grandchildren in providing both material and/or financial support and care. Together with those studies, this study provided more evidence of mutual care between grandparents and grandchildren in Vietnam. This finding also implies that care relationship, particularly between older persons and family care-givers, remains important in supplying continuous care for older persons (42).

Given limited and underdeveloped long-term care services for older people in both communities and institutions in Vietnam, such family care arrangements will continue to play a crucial role in providing care to older people who need assistance with ADLs. Socio-economic and demographic changes along with the shift in family values among generations in Vietnam, however, will be a key challenge for family care for older people as these factors will possibly change care provision in both care providers and patterns. For example, similar to the situation in other middle-income countries [see, for instance, (43) for Iran; (44) for China; and (7) also for China], Vietnam has a great shortage of systematic and organized training and education for home caregivers, and this has impaired the quality of care. Also, the burden of caregiving at home is hidden and biased to women, but they are not compensated by any social protection benefit scheme (45).

One of the most noticeable results from this study was that older people—regardless in urban or rural areas—living alone had the highest rate of ADL difficulty, but they had the lowest rate of receiving care from other persons. Such a situation also requires more attention in order to provide adequate care for this group of people.

## Limitations of this study

Although this study could provide evidence-based analyses for various policy implications in terms of family/home-based care for the Vietnamese older people, it obviously had some limitations. As the VNAS provided a cross-sectional data set, we could not detect causal inferences between dependent and independent variables. For example, we could not explain whether children provided care to their older parents was due to inheritance rather than filial piety. Similarly, it would have been more informative if VNAS could have provided data on caregivers' time use for care work, satisfaction of older people with their received care, cost of care at home, and how care influenced health of the older Vietnamese. Care provided by children living away from home should also be considered as an important source under rapid urbanization and migration.

## Data availability statement

The original contributions presented in the study are included in the article/supplementary material, further inquiries can be directed to the corresponding author: nttrang@isms.org.vn.

## Ethics statement

The studies involving human participants were reviewed and approved by the Institutional Review Board in Biomedical Research of the Institute of Social and Medical Studies (ISMS), Hanoi, Vietnam. The patients/participants provided their written informed consent to participate in this study.

## Author contributions

LG formed the research questions and writing ideas, calculated data, writing, and finalized the drafts. NN checked the data and provided comments. TP and PP supported in data processes and calculations. All authors contributed to the article and approved the submitted version.

## References

[1] United Nations' Economic and Social Commission for Asia and the Pacific (UNESCAP). Ageing in the Asian and Pacific Region: An Overview. Bangkok: UNESCAP (2017).

[2] UNFPA and HAI (HelpAge International). Ageing in the Twenty-First Century: A Celebration and a Challenge. London and New York: HAI and UNFPA (2012).

[3] United Nations' Department of Economic and Social Affairs (UNDESA). World Population Ageing 2017 (ST/ESA/SER.A/408). New York: UNDESA (2017).

[4] World Bank. Live long and prosper: Aging in Asia and Pacific. Washington DC: World Bank (2016).

[5] He W Weingartner RM Sayer LC. Subjective Well-Being of Eldercare Providers: 2012-2013. Washington DC: U.S. Census Bureau (2018).

[6] World Health Organization (WHO). World Report on Ageing and Health. Geneva: WHO (2015).

[7] Glinskya E Feng Z editors. Options for Aged Care in China—Building an Efficient and Sustainable Aged Care System. Washington, DC: World Bank (2018). 10.1596/978-1-4648-1075-6

[8] General Statistics Office Vietnam (GSO). Population and Housing Census 2019: Key Findings. Hanoi: GSO (2020).

[9] GSO. Population Projections for Vietnam, 2019–2069: Key Findings. Hanoi: Youth Publishing House (2020).

[10] Le MG. How can community organizations facilitate work in later life in ageing societies? in Presentation at the HelpAge Asia-Pacific Regional Conference ‘Family, Community and State in Ageing Societies' in Tehran, Iran, 23–25 October (2018).34468741

[11] Giang TL editor. Older Persons in Vietnam: Health Status, Utilization of Healthcare Services, and Policy Issues (in Vietnamese). Hanoi: National Economics University Publishing House (2020).

[12] MOH (Ministry of Health) and HPG (Health Partnership Group). Joint Annual Health Report 2016: Towards a Healthy Aging in Vietnam. Hanoi: Medical Publishing House (2018).

[13] Giang TL Pfau WD. Patterns and determinants of living arrangements for the elderly in Vietnam. In Giang TL, editors. Social Issues Under Economic Transformation and Integration in Vietnam, Volume 2. (2007), 147–76.

[14] Nguyen VC Tran TT. The impact of domestic remittances on the left-behind older people in Vietnam. J Econ Dev. (2018) 18:30–40. 10.33301/2016.18.03.02

[15] Giang TL Nguyen TT Nguyen NT. Social support and self-rated health among older men and women in Vietnam. J Popul Ageing. (2020) 13:427–42. 10.1007/s12062-020-09283-6

[16] Teerawichitchainan B Pothisiri W Giang TL. How do living arrangements and intergenerational support matter for psychological health of elderly parents? Evidence from Myanmar, Vietnam, and Thailand. Soc Sci Med. (2015) 136–137:106–116. 10.1016/j.socscimed.2015.05.019 25993521

[17] Tran TT. Impact of Domestic Migration on the Left-Behind Older People in Vietnam. (in Vietnamese) (Unpublished doctoral dissertation). National Economics University, Hanoi, Vietnam (2018).

[18] VWU. Vietnam Aging Survey 2011: Key Findings. Hanoi: Women's Publishing House (2012).

[19] O'Bien RM. A caution regarding rules of thumb for variance inflation factors. Qual Quant. (2007) 41:673–90. 10.1007/s11135-006-9018-6

[20] Archer KJ Lemeshow S. Goodness-of-fit test for a logistic regression model fitted using survey sample data. Stata J. (2006) 6:97–105. 10.1177/1536867X0600600106

[21] Giang TL Phi MP. Utilization and financial burden of healthcare services for older people in Vietnam (in Vietnamese). Econ Stud. (2017) 12:45–54.

[22] United Nations Population Fund (UNFPA). The Aging Population in Vietnam: Current Status, Prognosis, and Possible Policy Responses. Hanoi: UNFPA (2011).

[23] Vlachantoni A Shaw RJ Evandrou M Falkingham J. The determinants of receiving social care in later life in England. Ageing Soc. (2015) 15:321–45. 10.1017/S0144686X1300072X 25620821 PMC4298254

[24] Kemper P. The use of formal and informal home care by the disabled elderly. Health Serv Res. (1992) 27:421–51. 1399651 PMC1069888

[25] Hu B Ma S. Receipt of informal care in the Chinese older population. Ageing Soc. (2018) 38:766–93. 10.1017/S0144686X16001318

[26] Teerawichitchainan B Knodel J. Long-term care needs in the context of poverty and population aging: the case of older persons in Myanmar. J Cross Cult Gerontol. (2018) 33:143–62. 10.1007/s10823-017-9336-2 28988375

[27] Pickard L Wittenberg R Comas-Herrera A Davies B Darton RR. Relying on informal care in the new century? Informal care for elderly people in England to 2031. Ageing Soc. (2000) 20:745–72. 10.1017/S0144686X01007978

[28] Glaser K Stuchbury R Tomassini C Askham J. The long-term consequences of partnership dissolution for support in later life in the United Kingdom. Ageing Soc. (2008) 28:329–51. 10.1017/S0144686X07006642

[29] Phi MP Ho VH Luong TM Hoang TL. Unmet needs of care among older people in Vietnam. In *Proceedings of the Third Conference on Contemporary Issues in Economics, Management, and Business*. Hanoi: National Economics University (2020). p. 857–871.21321793

[30] Larsson K Silverstein M. The effects of marital and parental status on informal support and service utilisation: a study of older Swedes living alone. J Aging Stud. (2004) 18:231–44. 10.1016/j.jaging.2004.01.001

[31] Vyas S Kumaranayake L. Constructing socio-economic status indices: how to use principle component analysis. Health Policy Plan. (2006) 21:459–68. 10.1093/heapol/czl029 17030551

[32] Chow GC. Tests of equality between sets of coefficients in two linear regressions. Econometrica. (1960) 28:591–605. 10.2307/1910133

[33] Lee RL Dwyer JW Coward RT. Gender differences in parent care: demographic factors and same-gender preferences. J Gerontol B Psychol Sci Soc Sci. (1993) 48:S9–S16. 10.1093/geronj/48.1.S9 8418155

[34] Phillips DR Feng Z. Challenges for the aging family in the People's Republic of China. Can J Aging. (2015) 34:290–304. 10.1017/S0714980815000203 26300189

[35] Peng R Anstey KJ. Longitudinal study of factors associated with informal care provision: evidence from older Australians. Austral J Ageing. (2019) 2019:1–9. 10.1111/ajag.12613 30809886

[36] Utomo A McDonald P Utomo I Cahyadic N Sparrow R. Social engagement and the elderly in rural Indonesia. Soc Sci Med. (2018) 229:22–31. 10.1016/j.socscimed.2018.05.009 29754781

[37] Reiss IL Lee GR. Family Systems in America (4th ed.). New York: Holt, Rinehart and Winston (1988).

[38] Mujahid G. Population Ageing in East and South-East Asia: Current Situation and Emerging Challenges. Bangkok: UNFPA (2006).

[39] Abalos JB Saito Y Cruz GT Booth H. Who cares? Provision of care and assistance among older persons in the Philippines. J Aging Health. (2018) 30:1536–55. 10.1177/0898264318799219 30270711

[40] Giang TL Phi MP Ngo NV. Aging population, elderly care needs, and elderly care system in Vietnam: a snapshot. In: Presentation at the Joint World Bank-MOLISA (Ministry of Labour, War Invalids and Social Affairs) Working Group Meeting on Elderly Care System in Vietnam on 25 February 2019 at MOLISA (Hanoi, Vietnam) (2019).

[41] Giang TL Pham THT Phi MP. Productive activities of the older people in Vietnam. Soc Sci Med. (2019) 229:32–40. 10.1016/j.socscimed.2018.09.054 30301577

[42] Wilberforce M Challis D Davies L Kelly MP Roberts C Clarkson P. Person-centredness in the community care of older people: a literature-based concept synthesis. Int J Soc Welf. (2016) 26:86–98. 10.1111/ijsw.12221

[43] Amini R Chee KH Keya S Ingman SR. Elder care in Iran: a case with a unique demographic profile. J Aging Social Policy. (2020) 33:611–25. 10.1080/08959420.2020.1722896 31992153

[44] Cong Z Silverstein M. Parents' depressive symptoms and support from sons and daughters in rural China. Int J Soc Welf. (2011) 20:S4–S17. 10.1111/j.1468-2397.2011.00823.x 21984870 PMC3185374

[45] Institute of Labour Science and Social Affairs (ILSSA). An Overview of the Social Assistance Programs in Vietnam. A Background Paper for the MOLISA (Vietnam)—UNDP Project on Social Assistance Reform and Development. Boston: ILSSA (2016).

